# Particles of echovirus 18 open to release their genomes in vivo

**DOI:** 10.1073/pnas.2601182123

**Published:** 2026-07-20

**Authors:** Liya Mukhamedova, David Buchta, Zuzana Trebichalská, Yevgen Levdansky, Jana Moravcová, David Potěšil, Zbyněk Zdráhal, Dominik Hrebík, Lucie Nepovímová, Torleif Tollefsrud Gjølberg, Jan Terje Andersen, Jiří Nováček, Tibor Füzik, Pavel Plevka

**Affiliations:** ^a^Central European Institute of Technology, Masaryk University, Brno 62500, Czech Republic; ^b^ Department of Immunology, Oslo University Hospital Rikshospitalet, Oslo 0372, Norway; ^c^ Institute of Clinical Medicine and Department of Pharmacology, University of Oslo and Oslo University Hospital Rikshospitalet, Oslo 0372, Norway; ^d^ Precision Immunotherapy Alliance, University of Oslo, Oslo 0372, Norway

**Keywords:** enterovirus, genome release, uncoating, cryo-ET, cryo-EM

## Abstract

Enteroviruses cause a broad spectrum of human diseases, ranging from mild respiratory or gastrointestinal infections to severe neurological disorders such as aseptic meningitis and encephalitis. Enterovirus cell entry involves receptor-mediated endocytosis followed by destabilizing rearrangements of the virus capsid that enable genome release. However, the mechanism of enterovirus genome release has not been visualized in infected cells. Here, we used cryoelectron tomography and microscopy to image echovirus 18 (E18) entry into host cells and its interaction with the neonatal Fc receptor (FcRn). 30 min postinfection, endosomes and cytoplasm contained empty capsids missing one or several pentamers of capsid proteins, providing evidence that in vivo E18 releases its genome through capsid opening. In vitro, FcRn binding induced the expulsion of pocket factors from hydrophobic pockets in VP1, priming the virus for uncoating. The cryoelectron microscopy reconstruction of genome-containing particles of E18 inside infected cells did not reveal pocket factors, indicating that receptor binding triggers the same priming process during infection. We did not detect activated particles in infected cells, suggesting that these intermediates are short-lived and rapidly release their genomes in vivo. Our results identify capsid opening as the in vivo mechanism of echovirus 18 genome release, providing structural evidence for a process previously only inferred from in vitro experiments.

## Significance

Enteroviruses are important human pathogens; however, the mechanism by which their genome is released, a prerequisite for initiating infection, has remained unclear. Particles of enteroviruses are formed by RNA genomes protected by a surface layer of capsid proteins. Using cryoelectron microscopy imaging of infected cells, we show that capsids of an enterovirus, echovirus 18, open and release RNA through the loss of one to three pentamers of capsid proteins. We further demonstrate that binding of the virus to the neonatal Fc receptor primes the virus particles for genome release. The absence of detectable genome release intermediates in infected cells indicates that enterovirus genome release is rapid. These findings establish capsid opening as the physiological uncoating mechanism of echovirus 18.

Enteroviruses are a genus of small RNA viruses within the family *Picornaviridae* that cause a range of diseases, from mild respiratory infections to severe meningitis or myocarditis (1). Echovirus 18 (E18) causes meningitis and encephalitis in children (2). The high mutation rates and genetic diversity of enteroviruses pose a challenge to public health (1, 3).

Enterovirus capsids are built from 60 copies of major capsid proteins VP1, VP2, and VP3 forming a pseudo-T = 3 icosahedral shell (4, 5). In addition, 60 copies of the minor capsid protein VP4 are attached to the inner face of the capsid (4, 5). The virus protein 1 (VP1) of most enteroviruses forms a hydrophobic cavity containing a small molecule, called a pocket factor (6, 7). The capsid, approximately 30 nm in diameter, encloses a single-stranded, positive-sense RNA genome of about 7,500 nucleotides. Characteristic features of the surface of enterovirus particles are canyons—depressions encircling fivefold axes of capsid symmetry (5).

Enteroviruses enter cells by receptor-mediated endocytosis. Receptors utilized by enteroviruses to infect cells include decay-accelerating factor, intercellular adhesion molecule 1, low-density lipoprotein receptor, scavenger receptor class B member 2, P-selectin glycoprotein ligand 1, and several types of integrins (8, 9). However, the neonatal Fc receptor (FcRn) was shown to be a pan-enterovirus receptor (10–12). FcRn is a heterodimer formed by a transmembrane heavy chain and β2-microglobulin (13, 14). The extracellular part of the heavy chain contains domains α1, α2, and α3. β2-microglobulin and the α3-domain of the FcRn heavy chain have immunoglobulin-like folds (13, 14). The structures of echoviruses 6, 18, and 30 in complex with FcRn were determined using cryoelectron microscopy (11, 12, 15). The binding of FcRn to the echovirus canyons promotes the release of pocket factors, a prerequisite for the formation of activated particles, which are characterized by the absence of pocket factors and VP4, externalization of N termini of VP1, reduced contacts between pentamers of capsid proteins, expanded capsids, and changes in genome distribution (9, 11, 12, 15–23). Capsids of activated particles contain two types of pores. The pores between twofold and fivefold symmetry axes of the capsids enable externalization of the N termini of VP1 subunits (18, 20, 21). The pores surrounding the twofold axes of icosahedral symmetry of capsids were speculated to serve as the release sites for VP4 and the genome (18, 20, 21); however, a substantial enlargement of the pores would be required to enable the passage of genomic RNA. Cryo-EM studies of echoviruses 18 and 30 exposed to acidic pH in vitro indicate that RNA release is enabled by the opening of enterovirus capsids and removal of pentamers of capsid proteins (17, 24). However, the processes of genome release have not been structurally characterized in vivo.

In this study, we used cryoelectron microscopy (cryo-EM) and tomography (cryo-ET) to show that, at physiological temperature, FcRn binding to E18 promotes expulsion of the pocket factor, thereby priming the virus particle for uncoating. Genome-containing particles of E18 inside infected cells also lack pocket factors, the loss of which was probably induced by the receptor binding. The cell entry of E18 leads to the formation of empty capsids lacking pentamers of capsid proteins, providing evidence that capsid opening is the mechanism by which enterovirus genomes are released in vivo.

## Results and Discussion

### Binding of E18 to FcRn Induces Pocket Factor Expulsion.

**Fig. 1.**
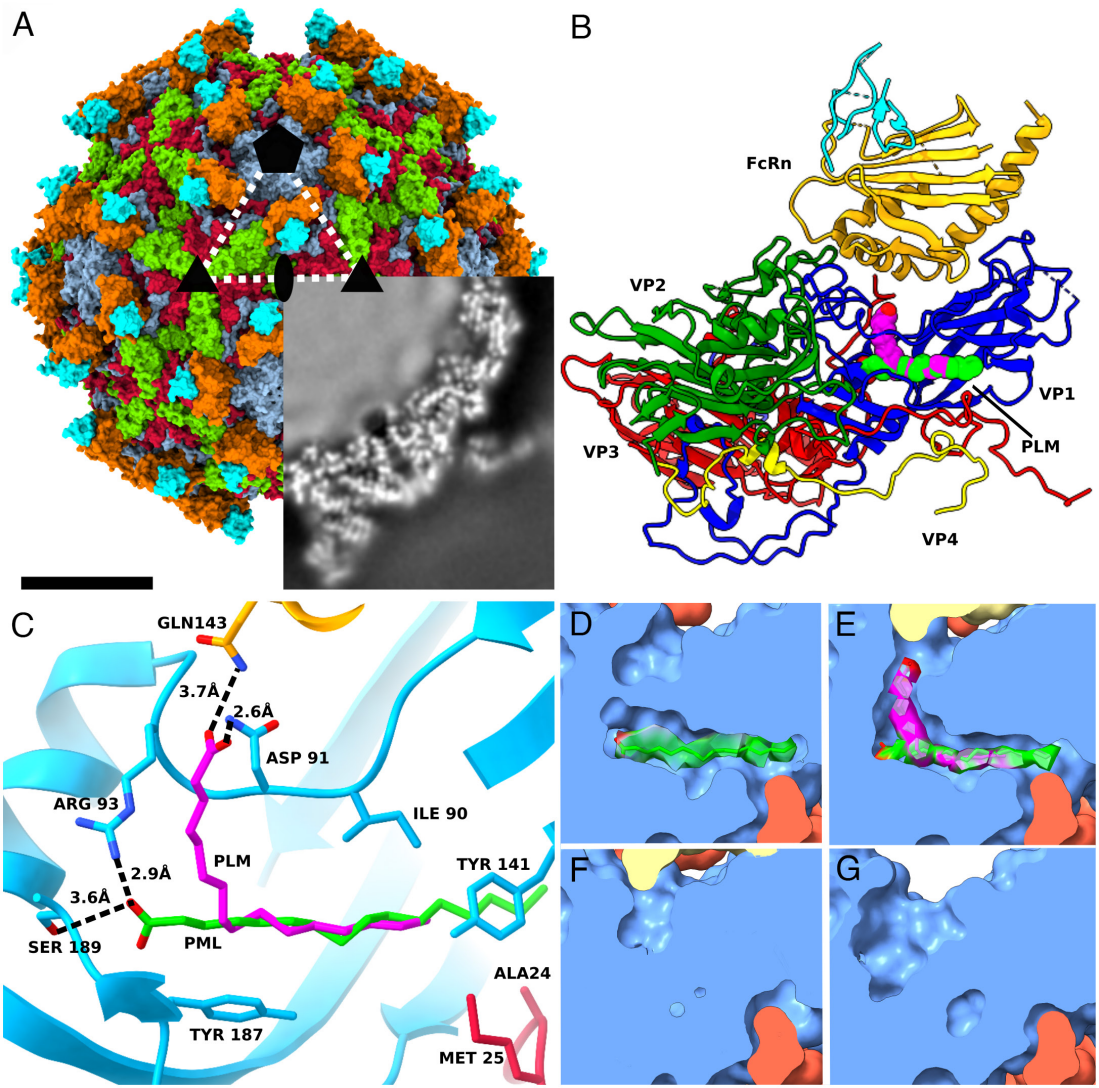
Binding of E18 to FcRn. (*A*) Molecular surface representation of E18 in complex with FcRn, color-coded to distinguish individual proteins. VP1 is shown in blue, VP2 in green, VP3 in red, and FcRn heavy chain and β2-microglobulins of FcRn in orange and cyan, respectively. The positions of selected icosahedral symmetry axes are indicated by a pentagon for fivefold, a triangle for threefold, and an oval for twofold. The dashed triangle indicates the border of a selected icosahedral asymmetric unit. The *Bottom* *Right* quadrant of the particle has been replaced by a central slice of cryo-EM density showing the presence of the RNA genome. White indicates high-density values. (*B*) Cartoon representation of the icosahedral asymmetric unit of E18 in complex with FcRn. The coloring is the same as in panel *A*. Alternate conformations of pocket factors in the VP1 pocket are shown in sphere representation. PLM indicates palmitic acid. (*C*) The complex of E18 with FcRn contains the pocket factor in two alternate conformations, corresponding to its position in the native virion (green) and in a partially expelled state (magenta). Proteins are shown in cartoon representation, and pocket factors and sidechains of selected residues interacting with the pocket factors in stick representation. The pocket is formed by residues of VP1 (blue) and closed on the inside of the capsid by two residues of VP3 from a neighboring icosahedral asymmetric unit (red). (*D*–*G*) Molecular surface representations of VP1 pockets in (*D*) E18 virion (PDB 6HBG), (*E*) E18 in complex with FcRn (PDB 9TF0), (*F*) E18 lacking the pocket factor in complex with FcRn (PDB 9TF0), and (*G*) an empty E18 capsid (PDB 9TF2). The images show cross-sections through the pockets with VP1 in blue, VP3 in red, and FcRn in light orange. Pocket factors in panels *D* and *E* are shown in stick representation. Cryo-EM densities of the pocket-factors are shown as semitransparent surfaces.

**Fig. 2.**
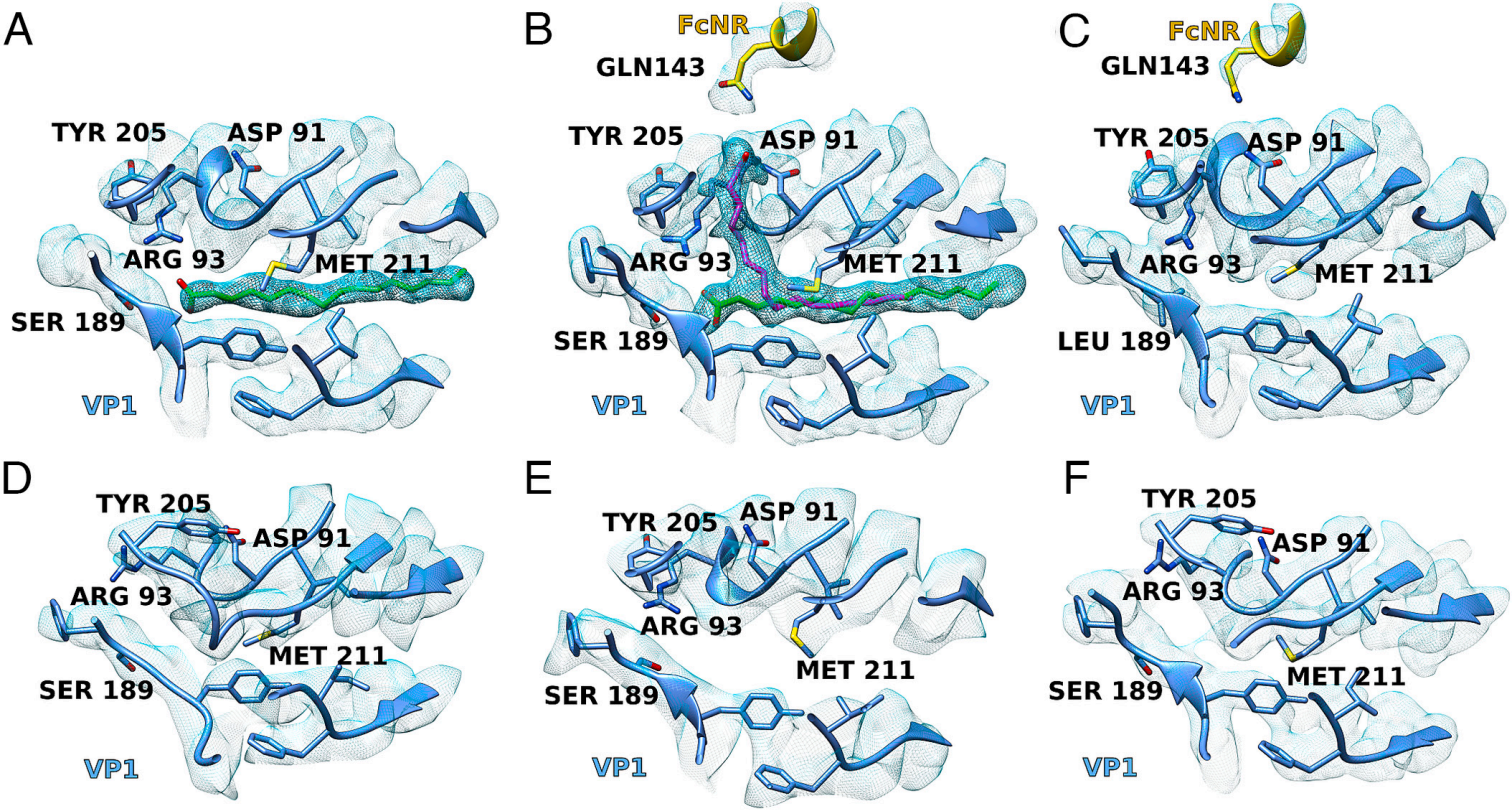
Comparison of VP1 pockets in E18 virion, E18–FcRn complexes, genome-containing particle in an infected cell, activated particle, and empty particle. Protein main chains are shown in cartoon representation and selected sidechains in stick representation. VP1 subunits are shown in blue and FcRn in yellow. Pocket factors are shown in stick representation in green and magenta. Electron density is depicted as a semitransparent blue mesh. (*A*) VP1 pocket of E18 virion contains pocket factor (PDB 6HBG). (*B*) VP1 pocket of E18–FcRn complex contains pocket factor in two alternate conformations (PDB 9TF0). (*C*) Alternate structure of the VP1 pocket of E18–FcRn complex lacking the pocket factor (PDB 7XXA). (*D*) Collapsed VP1 pocket of E18 activated particle (PDB 9TF1). (*E*) VP1 pocket of genome-containing particle in an infected cell lacking the pocket factor (PDB 9S63). (*F*) Collapsed VP1 pocket of E18 empty particle (PDB 9FT2).

The structures of echoviruses 6, 18, and 30 in complex with FcRn were determined previously (11, 12, 15). Our results corroborate that the binding site of E18 for FcRn is positioned in a canyon between the fivefold and twofold axes of icosahedral symmetry of the capsid (Fig. 1 *A* and *B* and *SI Appendix*, Fig. S1 and Table S1). Only the FcRn heavy chain interacts with the E18 capsid (Fig. 1*B*). The capsid structure of the E18 virion and that of the complex of the virus with FcRn can be superimposed with an RMSD of 0.49 Å for the corresponding Cα atoms, demonstrating that the binding of FcRn does not induce major conformational changes to capsid proteins (17). However, unlike the virions, the particles of E18 in complex with FcRn contain pocket factors in two positions (Fig. 1 *C*–*E*). The first position corresponds to that of a pocket factor fully buried in the VP1 pocket, as is the case in the virion (Figs. 1 *D* and *E* and 2 *A* and *B*). In the other position, the pocket factor is partially extruded from the hydrophobic pocket of VP1 (Figs. 1 *D* and *E* and 2 *A* and *B*). The fully buried conformation of the pocket factor is stabilized by hydrogen bonds between Arg93 and Ser189 of VP1 and the oxygen from the putative carboxyl group of the pocket factor (Fig. 1*C*). The putative carboxyl group of the partially extruded pocket factor forms hydrogen bonds with sidechains of Asp91 of VP1 and Gln143 of the FcRn heavy chain (Fig. 1*C*). Our observation of the partial pocket factor expulsion is consistent with a previous study of E18–FcRn complexes, in which it was shown that FcRn binding promotes the release of the pocket factor (Figs. 1*F* and 2*C*) (11, 15). The release of pocket factors from enterovirus capsids was suggested to precede the conversion of the virions to activated particles and genome release (9, 11, 12, 15–23).

### Genome-Containing E18 Particles in Cells.

**Fig. 3.**
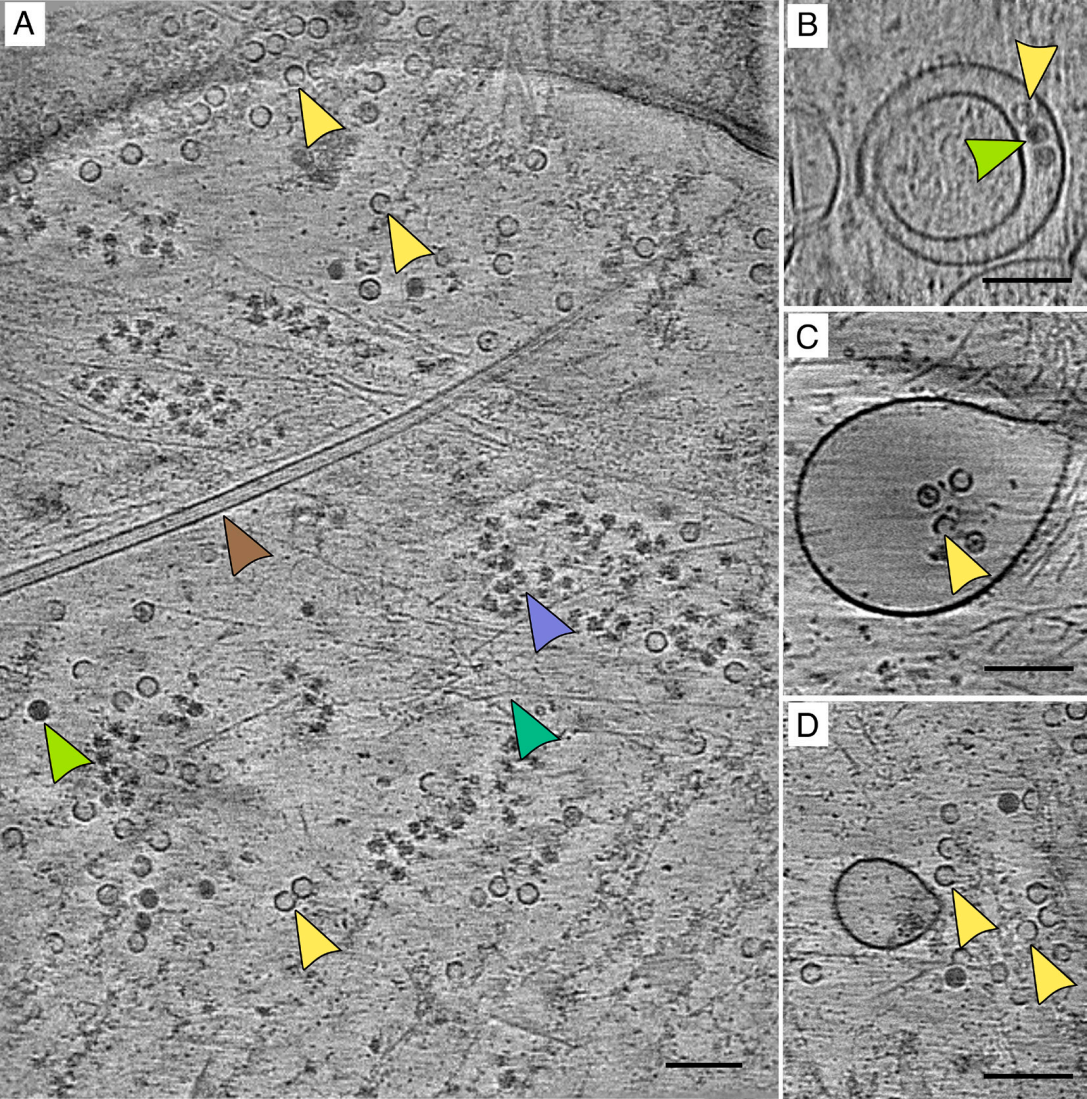
Cell entry and genome release of E18. Sections of cryotomograms of cos-7 cells 30 min postinfection. The cytoplasm (*A*) and vesicles (*B*–*D*) of the infected cells contain genome-filled and empty particles of E18. Yellow arrowheads indicate open capsids, green arrowheads indicate genome-containing particles, cyan arrowheads indicate actin filaments, the brown arrowhead indicates a microtubule, and the purple arrowhead indicates a ribosome. (Scale bar, 100 nm.)

**Fig. 4.**
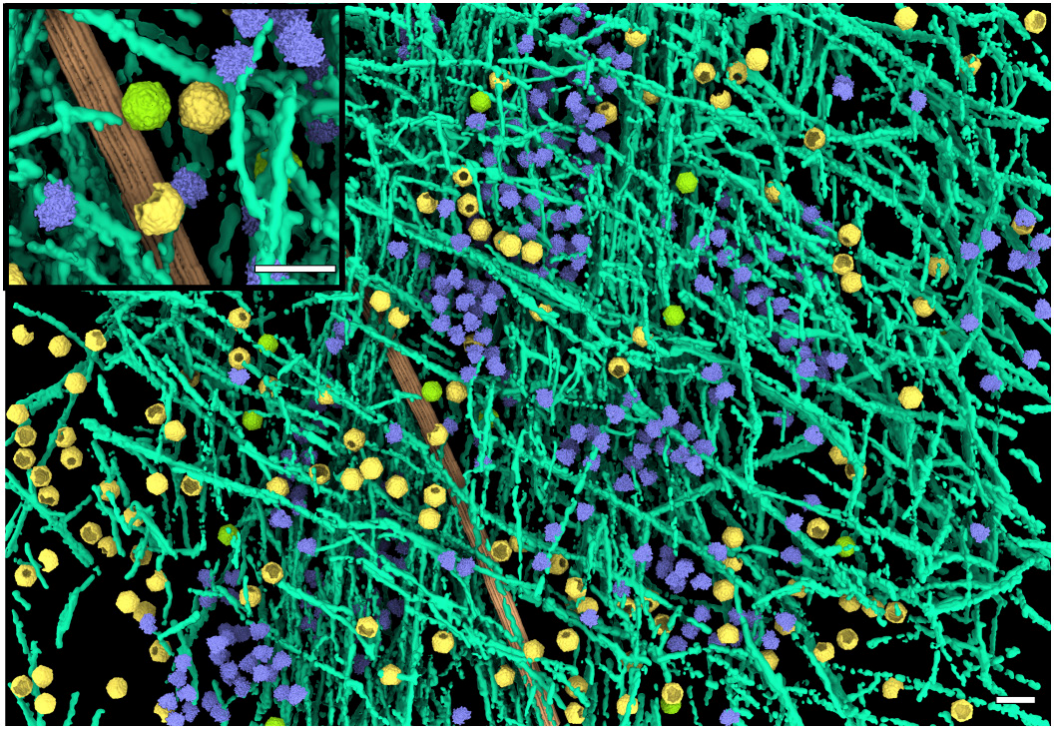
Segmentation of a cryotomogram showing the cytoplasm of a Cos7 cell 30 min postinfection by E18. The positions and orientations of genome-containing (green) and open E18 particles (yellow), as well as ribosomes (violet) and microtubules (brown), were determined using template matching. Actin filaments (cyan) were segmented manually. The magnified *Inset* in the *Upper* *Left* corner highlights the various particle states in the cell cytoplasm. (Scale bar, 50 nm.)

We employed cryo-EM and cryo-ET to visualize the entry of E18 into cos-7 cells, African green monkey kidney fibroblast-like cell line expressing SV40 large T antigen (Figs. 3 and 4, *SI Appendix*, Fig. S2, and Movie S1) (25). E18 inoculum used for the infection contained mostly virions with a minimal fraction of empty particles (*SI Appendix*, Fig. S3). Genome-containing particles of E18 attached to the cos-7 cell surface (*SI Appendix*, Fig. S4). The tomograms of the infected cells enabled the identification of genome-containing and empty E18 particles, ribosomes, microfilaments, microtubules, and cell membranes (Figs. 3 and 4 and Movie S1). As reported previously (26), 30 min postinfection, E18 particles were found in both membrane-bound vesicles and the cytoplasm (Fig. 3).

**Fig. 5.**
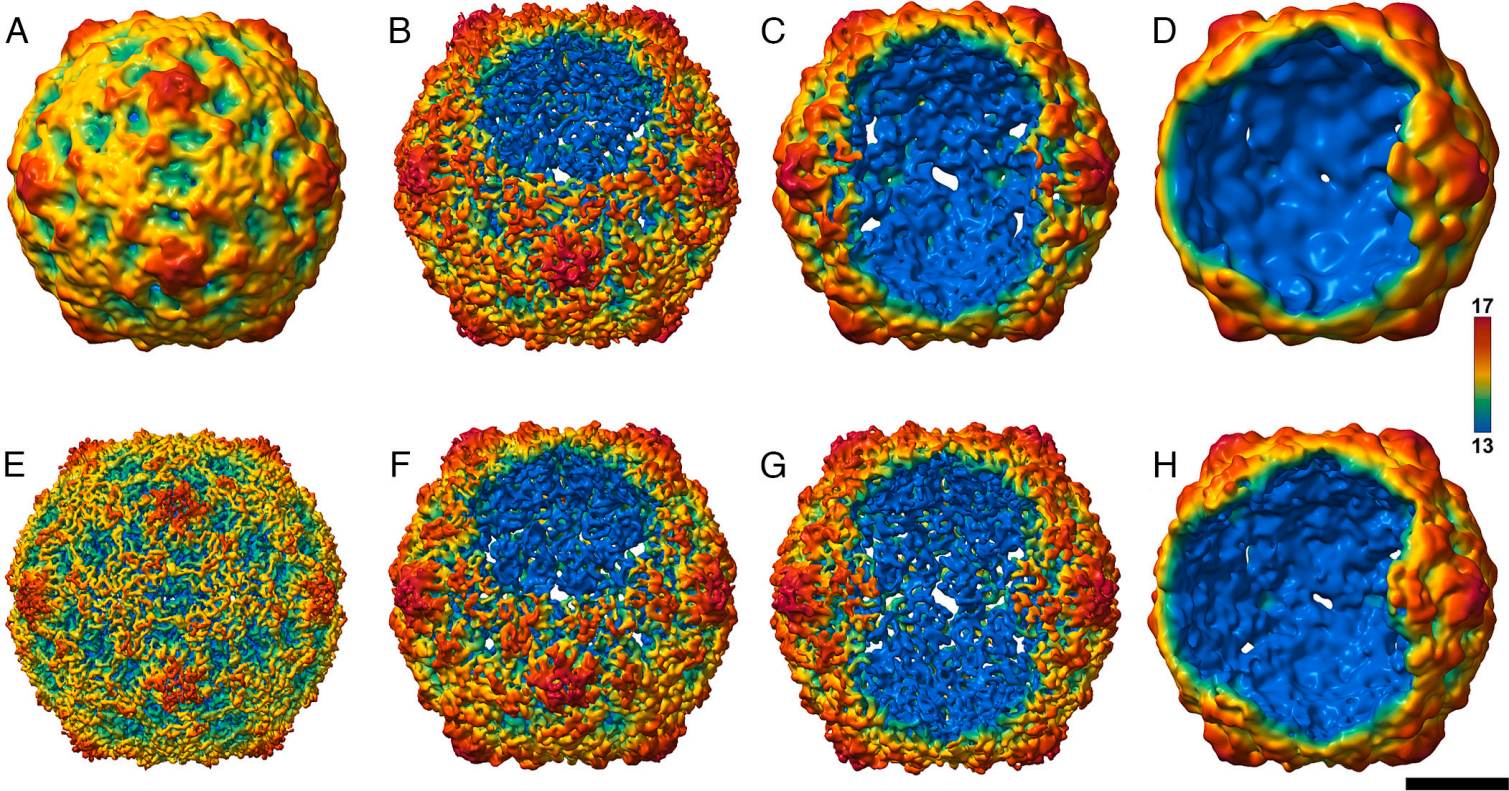
Cryo-EM reconstructions of E18 particles in infected cos-7 cells. The cryo-EM maps are shown in surface representation and rainbow colored based on the distance from the particle center. Asymmetric three-dimensional reconstructions of E18 genome-containing particle (*A*) and open particles lacking one (*B*), two (*C*), and three (*D*) pentamers of capsid proteins. Symmetrized three-dimensional reconstructions of genome-containing particle (*E*) and open particles missing one (*F*), two (*G*), and three (*H*) pentamers. (Scale bar, 10 nm.)

Single-particle reconstruction, using electron micrographs of infected cells, enabled the determination of the structure of E18 genome-containing particles to a resolution of 4.3 Å (Fig. 5 *A* and *E* and *SI Appendix*, Figs. S1 and S5 and Table S1). The structure was almost identical to that of a virion in vitro, with an RMSD of the corresponding Cα atoms of 0.88 Å (17). However, the reconstruction of the particles from cells lacks resolved density of pocket factors (Fig. 2*D*). We speculate that receptor binding or exposure to acidic pH in endosomes induced the pocket factor release. Similar to the structure of the E18–FcRn complex characterized in vitro, the VP1 pocket did not collapse (Figs. 1 *F* and *G* and 2 *C* and *D*). The collapse of the VP1 pocket in the absence of the pocket factor in E18–FcRn complexes is prevented by the sidechains of Met211 and Tyr205 of VP1 that partly fill the volume of the pocket (Fig. 2 *A*, *C*, and *D*) (15).

### Acidic pH Induces Detachment of E18 from FcRn.

Cryoelectron tomograms of infected cells show that the majority of E18 particles do not bind to the endosome membranes (Fig. 3 *B* and *C*) (17). It is likely that E18 particles entered cells by attaching to receptors, including FcRn, which is expressed by cos-7 cells (*SI Appendix*, Fig. S6), but the virus–receptor interactions were disrupted after endocytosis. Since the capsids of the genome-containing E18 particles in infected cells remain in a virion-like conformation, the detachment of the virus from receptors must be caused by conformational changes to the receptor or altered receptor–virus interactions. The FcRn interface includes the His196 of VP1, which is likely to change its protonation state upon endosome acidification (*SI Appendix*, Fig. S7). Furthermore, FcRn mediates the recycling of its ligands, IgG antibodies, and albumin, from endosomes to the extracellular space in both hematopoietic and nonhematopoietic cells (27, 28) and the transcytosis of the ligands across polarized epithelial cells that form the mucosal barriers (29, 30). Both of these processes occur in a pH-dependent manner, with the binding of IgG and albumin to FcRn at acidic pH and their release at neutral pH (30–32). Therefore changes in pH alter the properties of both the virus and receptor and may induce the disruption of E18–FcRn interactions in endosomes.

### Altered Particles of E18 were not Detected in Infected Cells.

Images of genome-containing E18 particles from infected cos-7 cells were subjected to extensive two-dimensional (2D) and three-dimensional (3D) classifications. Despite the demonstrated sensitivity of this approach for identifying a minor structural subset of virions among altered particles of CVA6 (33), we did not detect a subclass of E18 particles with altered capsids (Fig. 2*E*). As demonstrated in vitro for numerous enteroviruses, including E18, the formation of altered particles precedes genome release Fig. 2*F* and *SI Appendix*, Fig. S5 *C* and *D*) (26). In vitro, the transition of enterovirus virions to altered particles and subsequent genome release are induced by acidic pH (34–36). Here, we show that the treatment of cells with bafilomycin A1, a compound that prevents endosome acidification (37), resulted in accumulation of genome-containing particles in the endosomes and cytoplasm (*SI Appendix*, Fig. S8). The absence of altered particles in infected cells has been previously described for human rhinovirus 2 (26). It is likely that in vivo the altered E18 and rhinovirus 2 particles are short-lived intermediates that rapidly release their genomes and convert to empty capsids.

### E18 Releases its Genome by Capsid Opening.

Tomograms of infected cos-7 cells provide evidence that the empty particles of E18 have incomplete capsids (Figs. 3 and 4 and Movie S1). It has been previously shown that the genome release of E18 in vitro results in the formation of open particles missing up to three pentamers of capsid proteins (17). Single-particle reconstruction and 3D classification of images of empty particles from infected cells enabled the reconstruction of asymmetric and symmetrized structures of empty particles missing one, two, and three pentamers of capsid proteins (Fig. 5 *B*–*D* and *F–H* and *SI Appendix*, Fig. S1 and Table S1). Except for the missing pentamers of capsid proteins, the structures of the open particles corresponded to those of altered and empty particles, characterized by 3% radial expansion relative to the virion, reduced contacts among pentamers of capsid proteins, externalized N termini of VP1, and pores along twofold symmetry axes (9, 18–21, 38).

Despite extensive classification, we did not detect a subclass of empty particles with complete capsids in infected cells. Additionally, we attempted to identify the empty particles with complete capsids using CryoDRGN, an AI tool for the continuous reconstruction of heterogeneous cryo-EM datasets (39). This analysis resulted in three clusters: one composed of virions, another composed of particles missing one or two pentamers, and a third composed of particles missing three pentamers; however, no class of complete, empty capsids was detected (*SI Appendix*, Fig. S9). In contrast, a previous study of E18 genome release in vitro identified the presence of complete, empty capsids (17). However, the in vitro experiment involved high concentrations of purified virus particles in which the released pentamers could reattach to incomplete capsids (17). The putative reassociation under in vitro conditions was corroborated by computer simulations of genome release from particles mimicking the properties of enterovirus capsids (17, 40). The particles of E18 in infected cells are more dilute than in the in vitro experiments, and the open capsids and the released pentamers can interact with cellular components, which reduces the likelihood of their reassociation.

The activated particles of enteroviruses contain pores along twofold axes of icosahedral symmetry, which were proposed to serve for the release of VP4 subunits and genomes (18–22, 41–44). Furthermore, in vitro studies of poliovirus have indicated that the pores along the fivefold or twofold symmetry axes of the capsid may serve for genome release (19, 43, 45–47). However, asymmetric cryo-EM reconstructions of E18 and E30 particles exposed to acidic pH in vitro demonstrated that they form open particles missing pentamers of capsid proteins, and these openings may serve for genome release (17). Here, we show that most, if not all, empty particles of E18 in infected cells have incomplete capsids, providing evidence that capsid opening is the mechanism by which the E18 genome is released in vivo.

### E18 Capsid Opening in the Context of Genome Delivery to the Cytoplasm.

The delivery of enterovirus genomes across endosomal membranes has been extensively characterized through cellular and biochemical studies (48–52). Rhinoviruses use ICAM-1 or LDL receptors to attach to cells (53, 54). Minor group rhinoviruses, such as rhinovirus 2, were proposed to release their genomes via transmembrane pores formed by VP4 and the N termini of VP1 (20, 23, 55–57). Activated rhinovirus 2 particles were shown to bind to liposome membranes (55, 56, 58). In contrast, major group rhinoviruses and rhinovirus strains adapted to heparan sulfate have been shown to disrupt endosomal membranes and release their RNA into both endosomes and the cytoplasm (59, 60). The binding of poliovirus to CD155 induces the formation of activated particles, with externalized VP4 and the N termini of VP1, which have been shown to associate with liposome membranes in vitro (46, 61, 62). Furthermore, in vitro cryo-EM studies suggest that activated particles of poliovirus can release and translocate their RNA through the vesicle membrane via umbilical connections (45). Whereas poliovirus could employ a specific mechanism of genome delivery, it has been previously shown that neither the genome-containing nor empty particles of human rhinovirus 2, E18, E30, and EV71 associate with endosome membranes in vivo (26). Instead, genome-containing and empty particles of these viruses reach the cytoplasm via endosome disruption (*SI Appendix*, Fig. S10) (26). In agreement with previous studies, our results show that particles of E18 release their genomes both in endosomes and in the cytoplasm (Fig. 3) (26). It has been indicated that the cellular membrane remodeling mechanism disrupts enterovirus-containing endosomes (26). The endosome disruption process may involve galectin-8 and phospholipase PLA2G16, host factors that were shown to be essential for early enterovirus infection steps (63). Genome release by capsid opening is compatible with the delivery of E18 particles into the cell cytoplasm by endosome rupture (26).

## Conclusions

**Fig. 6.**
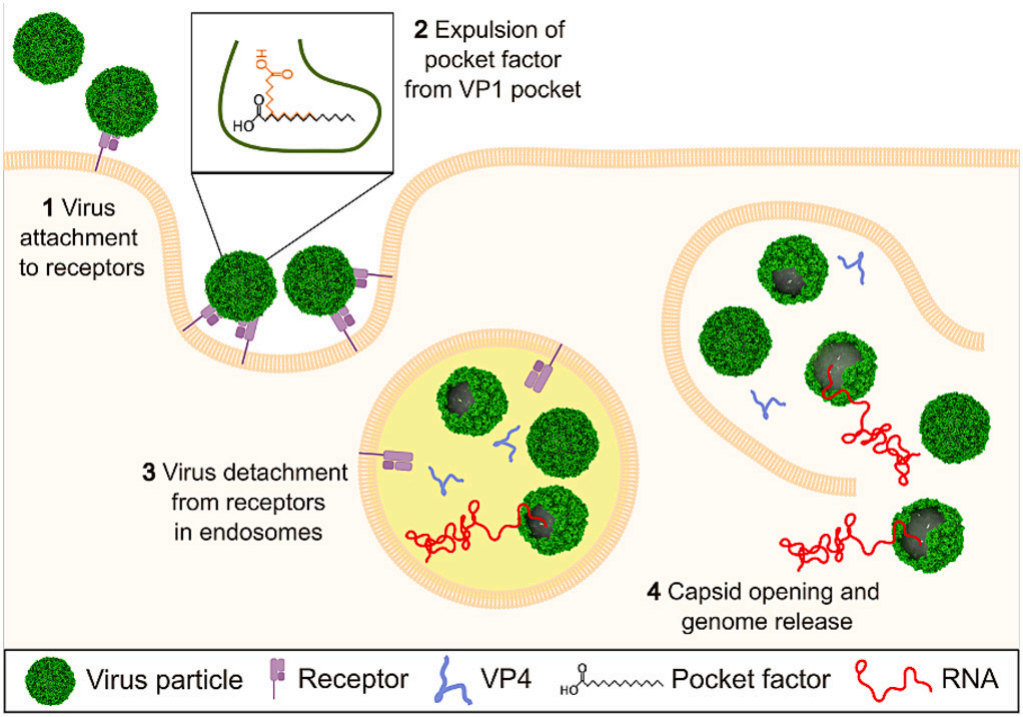
Overview of E18 cell entry. (1) E18 virions attach to FcRn at the cell surface and are internalized by endocytosis. (2) The receptor binding induces expulsion of pocket factors. (3) E18 particles dissociate from receptors in endosomes. The acidic pH triggers E18 genome release. (4) The membrane of some of the virus-containing endosomes ruptures, allowing virus particles to diffuse into the cytoplasm. Additional particles release their genomes into the cytoplasm. “Overview of E18 cell entry” by L. Mukhamedova created in BioRender (https://BioRender.com/yvhskbu), which is licensed under CC BY 4.0.

Enterovirus genome delivery is enabled by cell attachment, endocytosis, and genome release. The processes depend on virus–cell interactions that activate cellular processes and physically alter the virus particle. To gain entry into a host cell, the virions of E18 bind to FcRn (10–12), which triggers the expulsion of the pocket factors from VP1 pockets (Fig. 6). The receptor-bound particles are endocytosed. Inside endosomes, E18 particles dissociate from the receptors (Fig. 6). Since the genome-containing particles inside endosomes retain the same particle structure as virions, the detachment probably occurs due to the conformational changes to the receptor or alterations in the receptor–virus interactions in the acidic endosomal environment. Exposure to acidic pH promotes the activation of E18 particles in vitro (17). However, we did not detect the activated particles of E18 in infected cells, indicating that they rapidly release their genomes in vivo. All the empty particles in infected cells had incomplete capsids, missing one or several pentamers of capsid proteins, providing evidence that capsid opening is the mechanism of E18 genome release in vivo.

## Materials and Methods

### Virus Propagation.

E18 (strain METCALF, ATCC-VR-852) was propagated in GMK cells (ATTC-CCL-81) cultivated in Dulbecco’s modified Eagle’s medium enriched with 10% fetal bovine serum (Sigma, F7534). For virus preparation, 50 tissue culture dishes with a diameter of 150 mm, containing cells grown to 90% confluence, were infected with a virus inoculum at a multiplicity of infection of 0.01 to 0.1. The infection was carried out for 72 h, until 90% of the cells exhibited the cytopathic effect. The remaining attached cells were removed from the dishes using cell scrapers and harvested together with the floating cells and remaining media. The cell suspension was centrifuged at 15,300 × g in a Beckman Coulter Allegra 25R centrifuge, using a TA-10-250 rotor, at 10 °C for 30 min. The resulting pellet was resuspended in 10 mL of phosphate-buffered saline (PBS) (10 mM Na_2_HPO_4_, 1.8 mM KH_2_PO_4_, 137 mM NaCl, and 2.7 mM KCl, pH 7.4, Sigma-Aldrich). The solution was subjected to three rounds of freeze-thawing by transfer between −80 and 37 °C and homogenized using a Dounce tissue grinder. Cell debris was separated from the supernatant by centrifugation at 3,100 × g in a Beckman Coulter Allegra 25R centrifuge, using a TS-5.1-500 rotor, at 10 °C for 10 min. The resulting supernatant was combined with media from the infected cells. Virus particles were precipitated by the addition of PEG-8000 dissolved in PBS and NaCl to final concentrations of 5% (w/v) and 0.5 M, respectively. The precipitation was allowed to proceed at 10 °C with mild shaking (80 RPM) for 12 h. The following day, the precipitate was pelleted at 15,300 × g in a Beckman Coulter Allegra 25R centrifuge, using a TA-10 rotor, at 10 °C for 30 min. The supernatant was discarded, and the pelleted white precipitate was resuspended in 12 mL of PBS. MgCl_2_ was added to a final concentration of 5 mM, and the sample was subjected to DNAse (10 μg/mL final concentration) and RNAse (10 μg/mL final concentration) treatment for 30 min at ambient temperature. Subsequently, trypsin was added to a final concentration of 0.5 μg/mL, and the mixture was incubated at 37 °C for 10 min. pH 9.5 EDTA was added to a final concentration of 15 mM, and a nonionic detergent, NP-40™ (Sigma Aldrich Inc.), was added to a final concentration of 1%. The mixture was spun down at 3,100 × g in a Beckman Coulter Allegra 25R centrifuge, using an A-10 rotor, at 10 °C for 10 min. Particles from the resulting supernatant were pelleted through a 30% (w/v) sucrose cushion in resuspension buffer (0.25 M HEPES, pH = 7.5 and 0.25 M NaCl) by centrifugation at 210,000 × g in an Optima X 80 ultracentrifuge using a Beckman Coulter™ type 50.2 Ti rotor at 10 °C for 2 h. The pellet was resuspended in 1.5 mL of PBS and loaded onto 60% (w/w) CsCl solution in PBS. The CsCl gradient was established by ultracentrifugation at 160,000 × g in an Optima X80 ultracentrifuge using a Beckman Coulter™ SW41 Ti rotor at 10 °C for 18 h. The opaque band containing the virus was extracted with a 20-gauge needle mounted on a 5 mL disposable syringe. The virus was transferred into PBS by multiple rounds of buffer exchange using a centrifugal filter device with a 100-kDa molecular weight cutoff followed by buffer exchange using the gel filtration column from a DyEx 2.0 Spin kit (Qiagen).

### Plaque Assay.

RPE1b cells were cultured in Dulbecco’s Modified Eagle Medium (DMEM) supplemented with 10% fetal bovine serum (FBS) at 37 °C in a humidified 5% CO_2_ incubator. Cells were seeded into 6-well plates at a density of 7 × 10^5^ cells per well in 2 mL of medium (3.5 × 10^5^ cells/mL) and incubated overnight to allow attachment. Viral stocks were clarified by centrifugation at 5,000 × g for 1 min at 4 °C and filtration through a 20 µm pore filter. Serial 10-fold dilutions ranging from 10^–3^ to 10^–8^ were prepared in DMEM. For infection, cell monolayers were washed once with DMEM and inoculated with 400 µL of diluted virus solution per well. DMEM without FBS served as a negative control. After 2 h of incubation at 37 °C, the inoculum was removed and cells were overlaid with 2 mL of a mixture of MEM supplemented with 3% FBS and 5% low-melting-point agarose in a 3:2 ratio. Upon the stiffening of the agarose, an additional 1 mL of MEM supplemented with 3% FBS was added on top. Plates were incubated at 37 °C for 72 h to allow plaque formation. Cells were fixed with 10% formaldehyde in PBS for 2 h at room temperature, after which the monolayers were stained with 1% crystal violet in 20% ethanol for 15 min, washed, and dried.

### Preparation of Grids for Cryo-ET and Cryo-EM.

The grids with infected cells were prepared as described earlier (26). Briefly, 200 mesh holey carbon grids for transmission cryo-EM (Quantifoil, Au, R2/1) were sterilized by exposure to UV for 30 min and incubated in DMEM containing 10% fetal bovine serum (Sigma) for 15 min to increase cell adhesion. The grids were placed into Thermo Scientific Nunc Lab-Tek Chamber Slide cell culture dishes, and 80 µL of cos-7 cell suspension (2.5 × 10^5^ cells/mL in DMEM enriched with 10% fetal bovine serum) was added to each well and cultivated for 16 h. Cells were washed twice with PBS and infected with 5 × 10^10^ enterovirus particles in 5 µL of DMEM per well. The infection was carried out at 37 °C and 5% CO_2_ for 10 min. Subsequently, the virus suspension was removed, cells were washed with DMEM, fresh DMEM was added, and the cells were incubated at 37 °C and 5% CO_2_ for 20 min. Tomograms of E18-infected cells were used to determine the multiplicity of infection. The area of an average cos-7 cell was 2,500 μm^2^, and the area of a reconstructed tomogram was 4.4 μm^2^. There were 50 E18 particles in a tomogram of an infected cell. Therefore, an average cell was infected by about 29,000 virus particles. For enteroviruses, the ratio of infectious units to particles is approximately 1:1,000 (64–66). Therefore, the amount of virus attached to cells corresponded to a multiplicity of infection of 29, as described previously (26).

### Acquisition and Analysis of Cryoelectron Tomography Data.

Tomographic tilt series of infected cells were collected using a ThermoFisher Titan Krios transmission electron microscope operating at 300 kV. The data were collected using a post-GIF Gatan K3 (Bioquantum 967) direct electron detector operating in zero-loss imaging mode with the width of the energy selecting slit set to 20 e^−^V. The grid was first screened to localize cells with thin lamellipodia suitable for cryo-ET data collection. The tilt series were collected using SerialEM software (67) with a dose-symmetric tilt scheme (68) with an angular range of ±40° (2° increment) or ±60° (2.5° increment). With both settings, the total electron exposure was 190 e^−^/Å^2^. Individual images were saved as movies of eight frames in counting mode. The data were collected at a magnification of 33,000×, corresponding to a pixel size of 2.68 Å. Frames were aligned to compensate for drift and beam-induced motion during image acquisition using the software MotionCor2 (69). The resulting tilt series were aligned in IMOD using the patch tracking alignment algorithms, binned three times, and the tomograms were calculated by weighted back-projection with a SIRT-like filter as implemented in the software package IMOD (70).

Aligned tilt series from IMOD were imported into an emClarity work environment, and the contrast transfer function (CTF) of the tilts was determined using emClarity (v 1.5.3.11) (71). CTF-corrected tilt series were binned three times and reconstructed into tomograms. Template matching was performed by emClarity using resampled maps of ribosomes (EMD-8345), microtubules (EMD-12639), and maps of the E18 virions and open E18 capsids (17, 72, 73). The set of template-matched particles was curated by manual inspection of each matched particle’s position in the tomogram. Positions and orientations of the matched particles were exported from emClarity into a set of homemade scripts (https://github.com/fuzikt/tomostarpy; placeback_subvolume.py), which were used to position the maps of the search templates into a volume that matched the size of the original tomogram. The actin filaments were manually segmented in Dragonfly software (version 2022.2.0.12227, ORS) and exported as three-dimensional maps in MRC format (74). The maps of the positioned components were overlaid in ChimeraX (75) together with volumes of manually segmented membranes, actin filaments, and the original tomogram.

### Acquisition and Analysis of Single-Particle Data In Vivo.

Images of E18 infecting cos7 cells were collected using a ThermoFisher Titan Krios transmission electron microscope operating at 300 kV. The data were collected using a post-GIF Gatan K3 (Bioquantum 967) direct electron detector operating in zero-loss imaging mode with the width of the energy filter set to 20 e^−^V. Electron micrographs were recorded in the counting mode of the detector at a magnification of 42,000×, which corresponds to a pixel size of 2.08047 Å. Total electron exposure was 60 e^−^/Å^2^, and defocus values ranged from −2.5 to −4.5 μm. Movies of 40 frames were recorded for each 8 s of exposure. The frames from each exposure were aligned to compensate for drift and beam-induced motion using the software MotionCor2 (69). The resulting dose-weighted sums of aligned frames were used in the subsequent image processing steps, except for estimating CTF parameters, which were determined from non-dose-weighted micrographs using the program gCTF v1.06 (76). From 1,290 micrographs, 24,805 particles were initially boxed using CryoSparc Blob Tuner (CryoSPARC v 4.6.2, Structura Biotechnology Inc.) (77). Subsequent 2D classification reduced the dataset to 14,767 images, which were further classified into subsets: 1,337 genome-filled virions and 9,994 empty capsids. 3D classification of the empty capsids revealed structural heterogeneity. Heterogeneous refinement using six initial models based on prior knowledge of in situ genome release structures split the empty particles into four major classes: capsids with two openings on the opposite sides, and capsids missing one, two, or three pentamers. The class of capsids with two openings on the opposite sides was merged with the class of particles missing one pentamer for further 3D classification, which separated the particles into classes of capsids with one opening of various sizes. Overall, the dataset contained capsids missing one pentamer (3,896 particles), two pentamers (6,365 particles), three pentamers (2,114 particles), and genome-filled capsids (1,337 particles). CryoSparc homogeneous refinement of all structures was performed, with and without symmetry enforcement. The final resolutions of the maps were estimated using the gold-standard Fourier Shell Correlation approach.

### Preparation of E18 in Complex with the Receptor.

Recombinant soluble truncated human FcRn was produced and purified as described previously (78). The complex of E18 with FcRn was prepared by mixing purified virions of E18 at a concentration of 2 mg/mL with FcRn in a molar ratio of three receptors for each icosahedral asymmetric unit of the virus. The mixture was incubated on ice for 10 min and immediately vitrified on grids for electron microscopy, as described below.

### Acquisition and Analysis of Single-Particle Data for E18 in Complex with FcRn.

For cryo-EM, 3.5 μL of the E18–FcRn mixture was applied onto a holey carbon grid (Quantifoil R2/1 300 mesh), blotted, and vitrified using a Vitrobot Mark IV. Electron micrographs of virus particles in complex with the receptor were collected using a Titan Krios transmission electron microscope operating at 300 kV. Images were recorded with a Falcon III direct electron detection camera under low-dose conditions (48 e^−^/Å^2^) with defocus values ranging from −1.0 to −3.0 μm at a nominal magnification of 75,000×, corresponding to a pixel size of 1.061 Å/px. Each exposure (1 s) was recorded in movie mode and saved as 39 separate movie frames. The frames from each exposure were aligned to compensate for drift and beam-induced motion during image acquisition using the software MotionCor2 (69). The resulting dose-weighted sum of aligned frames was used in the subsequent image processing steps, except for the estimation of CTF parameters, which were determined from non-dose-weighted micrographs using the program gCTF (76). Particle images (512 × 512 pixels) were automatically picked from micrographs using the program Gautomatch. The images were processed using the package RELION (79). An electron density map calculated from the structure of echovirus 7 (PDB ID: 2X5I), low-pass filtered to a resolution of 50 Å, served as the initial model for 3D classifications (80). The volumes resulting from the 3D refinement were threshold masked, detector modulation transfer function corrected, and B-factor sharpened using the RELION postprocess procedure (79). The resolution of the reconstruction was estimated according to the FSC 0.143 criterion.

### Analysis of E18 Particle Heterogeneity in Infected Cells.

In order to analyze the structural heterogeneity of E18 capsids in situ, the software CryoDRGN (version 3.4.2) was used (39, 81). The volume decoder network was trained on the particle alignments obtained from C1 homogeneous refinements in cryoSPARC (77). The 10-D latent variable model was trained on the in situ E18 particle images. Due to the size of the reconstructed particles and their heterogeneity, the training and subsequent analysis were performed on twice-binned data. The 10-D latent space representation was projected to 2D using principal component analysis and uniform manifold approximation. In order to visualize the discrete conformational states of the E18 capsid, CryoDRGN conformational landscape analysis was performed. The parameters of the analysis included applying a mask to the capsid in order to subtract the noise from in situ images and reducing the number of generated volumes to 50.

### Preparation of Cryo-EM Samples of Cos-7 Cells Treated with Bafilomycin A1 and Infected by E18.

The preparation of samples to study the effects of bafilomycin A1 differed from that of normal infection only in the application of 200 nM for bafilomycin A1 in 5 µL of DMEM to cos7 cells grown on grids for 30 min. Subsequently, the medium covering the cells was replaced with 5 μL of DMEM containing E18 and 200 nM bafilomycin A1. After 30 min the cells were washed three times with PBS and vitrified, as described above.

### Mass Spectrometry Detection of NeoFc Expression in Cos7 Cells.

The cell pellet was lysed for 30 min at 95 °C using 0.1% n-Dodecyl β-D-maltoside in 50 mM ammonium bicarbonate buffer. Approximately 10 μg of total protein (protein concentration was estimated using quality control 1D SDS–PAGE gel and external calibration using protein lysate of known protein concertation) was used for the in-solution tryptic digestion (4 h at 37 °C) after the reduction (5 mM dithiothreitol, 30 min at 56 °C) and alkylation step (10 mM iodoacetamide, 30 min at laboratory temperature in the dark). The resulting peptide mixture was transferred into an LC–MS vial using 2.5% formic acid in 50% acetonitrile, then 2.5% formic acid in 100% acetonitrile. The solution volume was reduced while removing acetonitrile using SpeedVac (Thermo Fisher Scientific). Peptide concentration was estimated using quality control LC–MS analyses using RSLCnano (Thermo Fisher Scientific) online connected to an Impact II (Bruker) and an external calibration curve using HeLa digest of known peptide concentration (Pierce) and area under the total ion chromatogram as the peptide quantity measure.

LC–MS/MS analyses of the peptide mixture were performed using an UltiMate 3000 RSLCnano system (Thermo Fisher Scientific) connected to a timsTOF Pro HT mass spectrometer (Bruker). Before LC separation, tryptic digests were online concentrated and desalted using a trapping column (300 μm × 5 mm, μPrecolumn, 5 μm particles, PepMap™ Neo Trap Cartridge, Thermo Fisher Scientific). The trap column was then washed with 0.1% trifluoroacetic acid and the peptides were eluted in backflush mode from the trapping column into an analytical column (Aurora C18, 75 μm ID, 250 mm long, 1.7 μm particles, PN AUR3-25075C18-CSI; Ion Opticks) by a linear 90 min gradient program (flow rate 150 nL.min-1, 3 to 42% of mobile phase B; mobile phase A: 0.1% formic acid in water; mobile phase B: 0.1% formic acid in 80% acetonitrile) followed by a system wash using 80% of mobile phase B. Equilibration of the trapping column and the analytical column was done before sample injection to the sample loop. The analytical column was installed in the Captive Spray ion source (Bruker; temperatures set to 50 °C) according to the manufacturer’s instructions. The spray voltage was set to 1.4 kV.

Multistage mass spectrometry data were acquired in two modes. The first was a data-independent acquisition (DIA, diaPASEF) mode with an m/z range of 100 to 1,700 and 1/k0 range of 0.6 to 1.4 V s cm^−2^. Second-stage spectra were recorded from the m/z 400 to 1,000 precursor range using 30 windows and two steps for each PASEF scan, and a cycle time of 100 ms locked to a 100% duty cycle. Next, gas phase fractionation was utilized using narrow and overlapping ion mobility ranges in combination with narrow m/z ranges covered using the diaPASEF approach in five analyses in total. DIA data were processed using DIA-NN (version 2.1.0) (82) in library-free mode against an indexed database of the retention times of peptides and trypsin for *Chlorocebus sabaeus*, the source organism of cos-7 cells, (version from 2025-06-19, number of protein sequences: 19,142). During the library preparation, carbamidomethylation was set as a fixed modification, trypsin/P enzyme with one allowed missed cleavage, and peptide length 6 to 30. False discovery rate control was set to 1%. The accuracies and scan window parameters of the first and second stage mass spectrometry analyses were set to 15 ppm and 10 scans, respectively. The Match Between Runs option was switched on.

## Supplementary Material

Appendix 01 (PDF)

Movie S1. **Cryo-tomogram and segmentation together with template matching of the cytoplasm of a Cos7 cell, 30 minutes post-infection by E18**. The positions and orientations of genome-containing (green) and open E18 particles (yellow), as well as ribosomes (violet) and microtubules (brown), were determined using template matching. Actin filaments (cyan) were segmented manually. The magnified inset in the upper left corner highlights the various particle states in the cell cytoplasm. Scale bars represent 50 nm. The movie corresponds to the tomogram segmentation shown in Fig. 4.

## Data Availability

Cryo-EM density maps have been deposited in the Electron Microscopy Data Bank, https://www.ebi.ac.uk/pdbe/emdb/ [Accession Numbers EMD-54484 (83), EMD-54622 (84), EMD-54764 (85), EMD-55096 (86), EMD-54510 (87), EMD-54506 (88), EMD-54765 (89), EMD-54763 (90), EMD-55863 (91), EMD-55864 (92), and EMD-55865 (93)], and the atom coordinates have been deposited in the Protein Data Bank, www.pdb.org [PDB ID codes 9S63 (94), 9SPU (95), 9TF0 (96), 9TF1 (97), and 9TF2 (98)]. Electron micrographs and an example tomogram of E18 infected cells were deposited to EMPAIR under the accession code EMPIAR-13259 (99) and EMPIAR-13199 (100), respectively. Mass spectrometry proteomics data were deposited to the ProteomeXchange Consortium via PRIDE (101) partner repository under dataset identifier PXD068859 (102). The additional data that support the findings of this study are available from the corresponding authors upon request.
