# Supplementary material for: Particles of echovirus 18 open to release their genomes in vivo
Source: Proc Natl Acad Sci U S A. 2026 Jul 20;123(30):e2601182123. doi: 10.1073/pnas.2601182123 (PMC13416445; doi:10.1073/pnas.2601182123)
Supplement: Supplementary file 1 — Appendix 01 (PDF) [file pnas.2601182123.sapp.pdf]

Supplementary Information for:

Particles of echovirus 18 open to release their genomes *in vivo*

Short title: Enterovirus particles open to release their genomes

Liya Mukhamedova[1], David Buchta[1], Zuzana Trebichalská[1], Yevgen Levdansky[1$], Jana Moravcová[1], David Potěšil[1], Zbyněk Zdráhal[1], Dominik Hrebík[1], Lucie Nepovímová[1], Torleif Tollefsrud Gjølberg[2,3,4], Jan Terje Andersen[2,3,4], Jiří Nováček[1], Tibor Fuzik[1], Pavel Plevka[1&]

1 – Central European Institute of Technology, Masaryk University, Kamenice 5, Brno 62500, Czech Republic
2 – Department of Immunology, Oslo University Hospital Rikshospitalet, 0372, Oslo, Norway.
3 – Institute of Clinical Medicine and Department of Pharmacology, University of Oslo and Oslo University Hospital Rikshospitalet, 0372, Oslo, Norway.
4 – Precision Immunotherapy Alliance (PRIMA), University of Oslo, 0372, Oslo, Norway.

$ – Current address: Messenger RNA Regulation and Decay Section, RNA Biology Laboratory, Center for Cancer Research, National Cancer Institute, Frederick, MD, U.S.A.

& - Corresponding author
Address: Pavel Plevka, Laboratory of Structural Virology, Central European Institute of Technology, Masaryk University, Kamenice 753/5, 62500 Brno, Czech Republic
E-mail: pavel.plevka@ceitec.muni.cz
Telephone: +420 549 49 7756

Supplementary figures

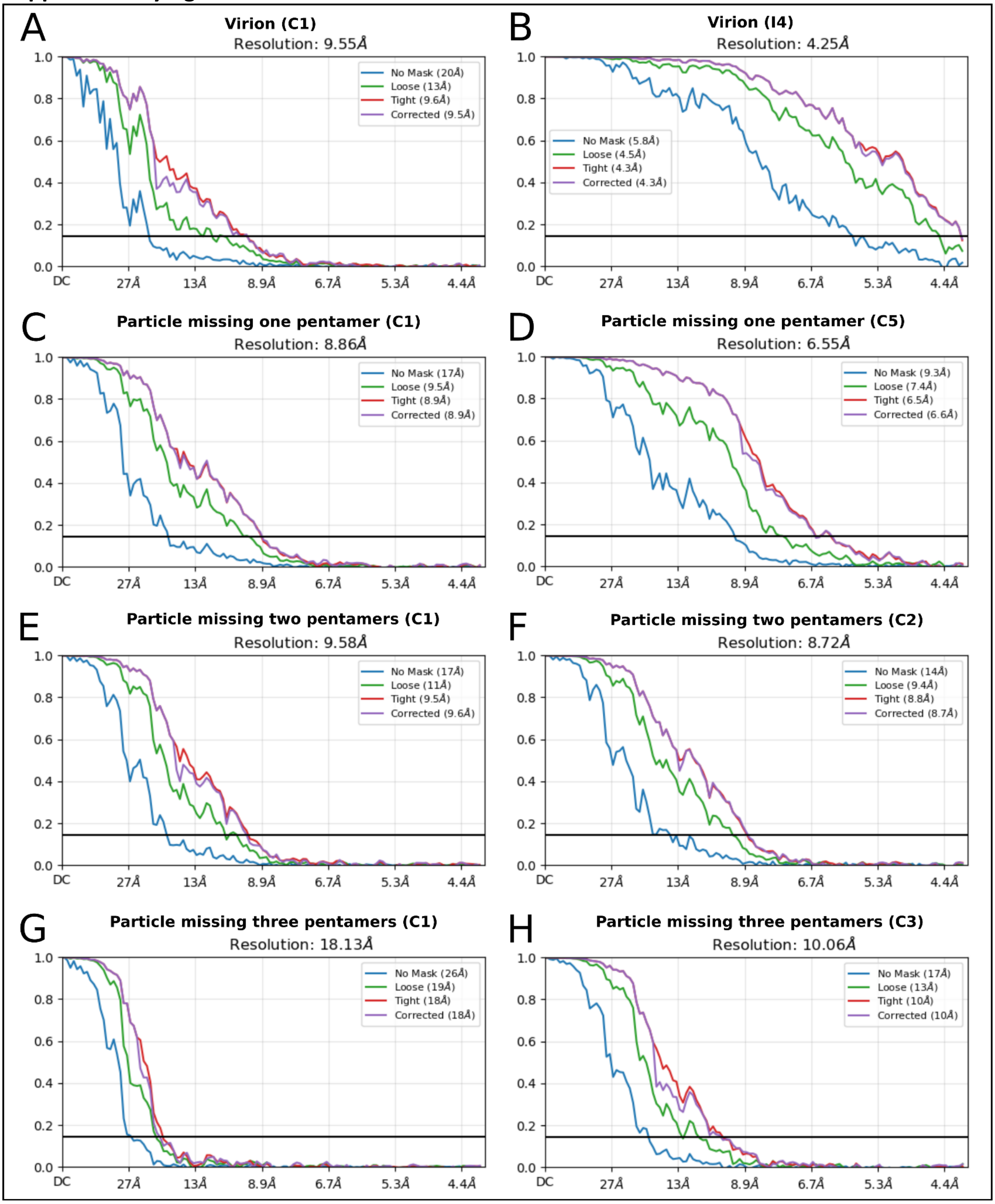

Fig. S1. FSC curves of cryo-EM reconstructions. The final resolution is reported for the FSC cutoff at 0.143.

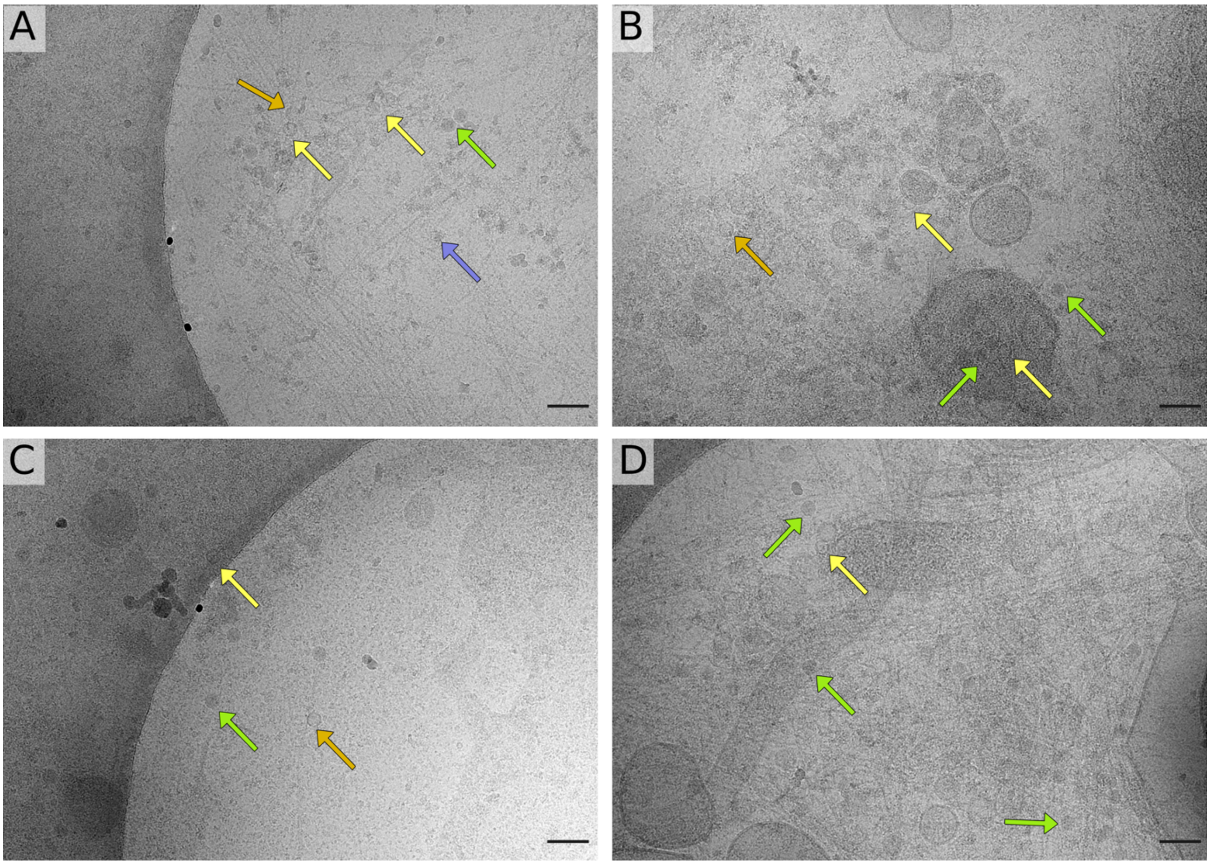

Fig. S2. Example electron micrographs of E18-infected cos-7 cells 30 minutes post-infection. The genome-containing particles are indicated by green arrows, empty particles by yellow arrows, open particles by orange arrows, and ribosomes by violet arrows. Scale bars indicate 100 nm.

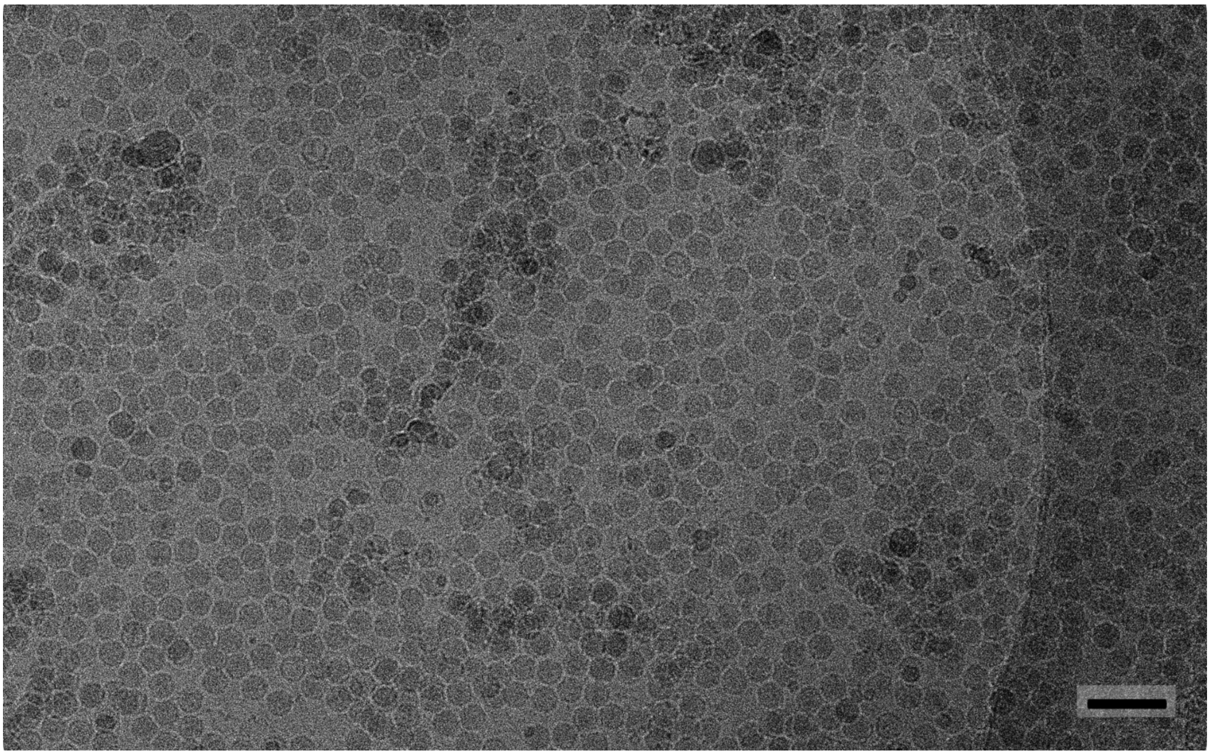

Fig. S3. Electron micrographs of E18 inoculum used to infect cos-7 cells. Scale bar indicates 100 nm.

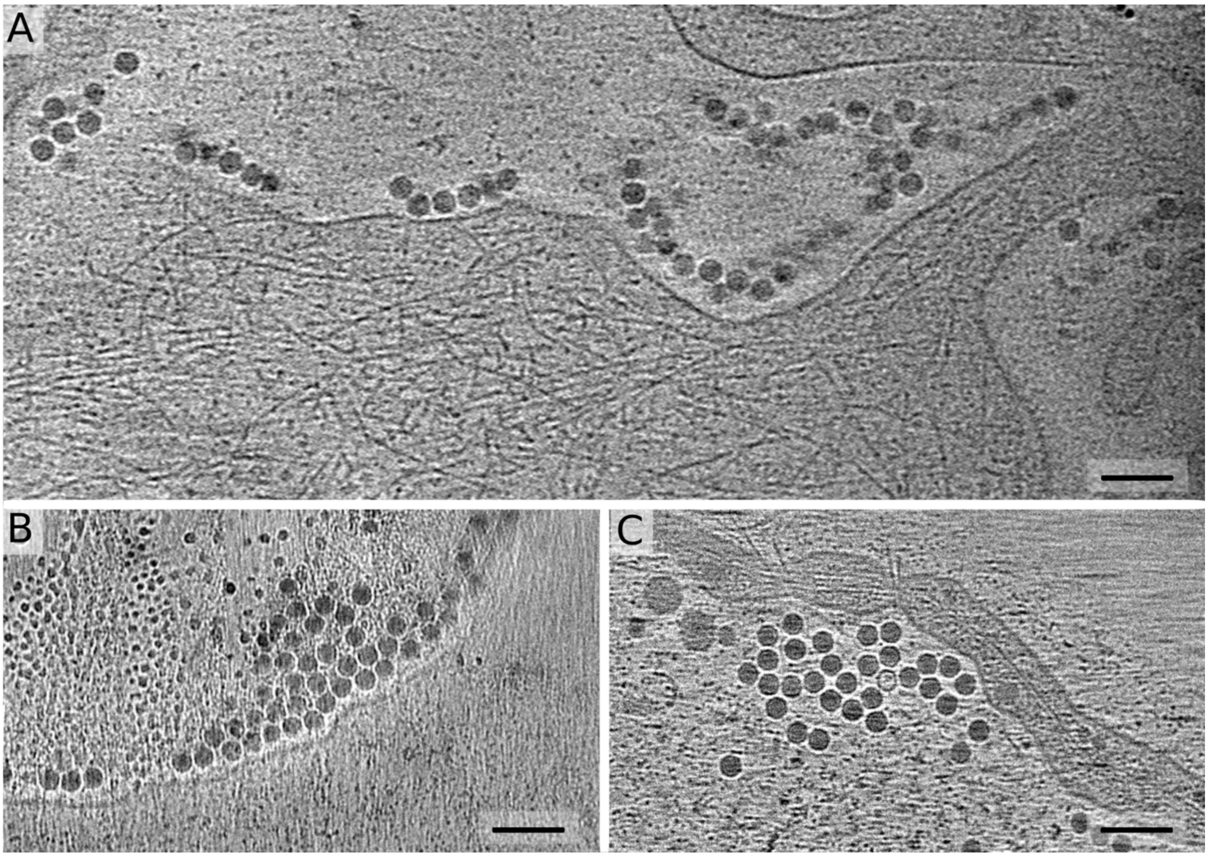

Fig. S4. Cell attachment of E18. (A-C) Sections of cryo-tomograms of cos-7 cells showing attachment of E18 particles to the cytoplasmic membrane. Scale bars indicate 100 nm.

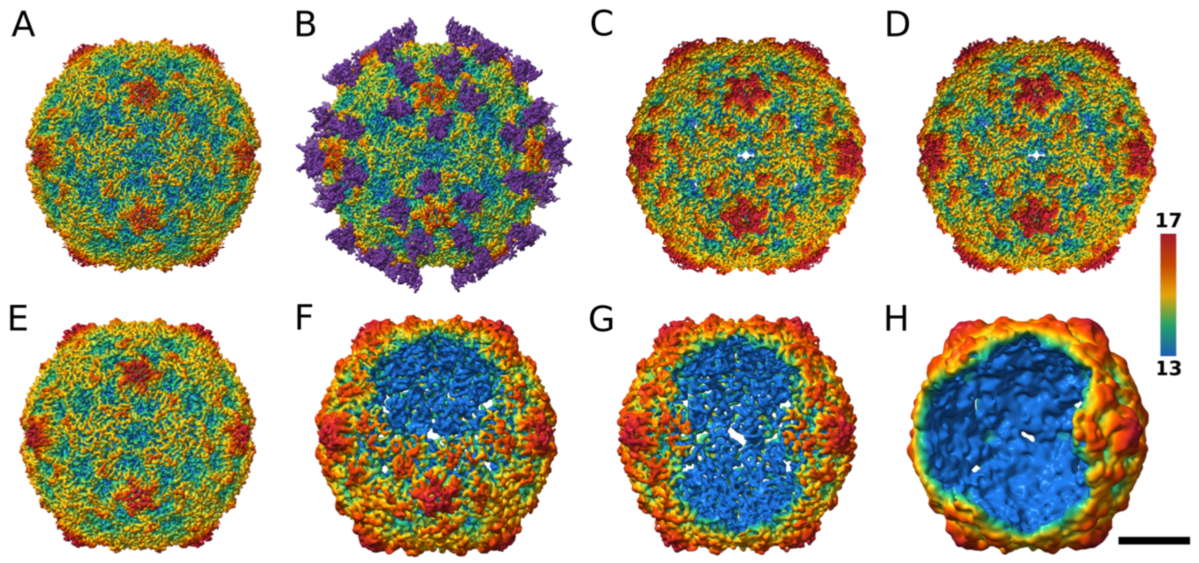

Fig. S5. Comparison of cryo-EM reconstructions of various types of E18 particles. Surface representations of cryo-EM reconstructions of: **(A)** E18 virion determined *in vitro*. **(B)** E18 in complex with FcRn determined *in vitro*. FcRn are shown in magenta. **(C)** Activated particle determined *in vitro.* **(D)** An empty particle determined *in vitro.* **(E)** Genome-containing particle determined *in situ.* **(F-H)** Open particles missing one (F), two (G), and three (H) pentamers of capsid proteins determined *in situ*. The maps are rainbow-colored from blue to red, based on the increasing distance of the surface from the particle center. Scale bar indicates 10 nm.

Seq.	Prec. Count (sample)	Prec. Count (GPF1)	Prec. Count (GPF2)	Prec. Count (GPF3)	Prec. Count (GPF4)	Lib.Q Value	PEP (sample)	PEP (GPF1)	PEP (GPF2)	PEP (GPF3)	PEP (GPF4)
1	1		1	1		3.93E-07	5.60E-05		5.94E-07	1.33E-03	
2	2	1	1	1	1	4.96E-06	1.56E-03	2.25E-03	5.11E-03	1.81E-04	2.37E-04
3	1			1	1	5.98E-07	1.56E-03			1.33E-03	2.37E-04
4	1			1	1	8.81E-05	1.80E-04			1.81E-04	2.37E-04
5			1	1	1	1.38E-04			2.75E-03	1.33E-03	2.37E-04
6			1	1	1	7.97E-04			9.69E-02	5.55E-03	3.32E-03

	Sequence
1	SGLPAPWISLR
2	ELTFLLFSCPHR
3	GDDTGSLLPTPGEAQDADSK
4	QGTWGGDWPEALAISQR
5	ARPGNPGFSVLTCSAFSFYPPELQLR
6	GDDTGSLLPTPGEAQDADSKDINVIPATA

Sequence coverage

SLSAESHLSLLYHLTAVSSPAPGTPAFWVSGWLGPQQYLSYDSLRGQAEPCGAWVWENQVSWYWEKETTDLRIKEKLFLEAFKALGGK
GPYTLQGLLGCELSPDNTSVPTAKFALNGEEFMNFDHKQGTWGGDWPEALAISQRWQQQDKAANKELTFLLFSCPHRLREHLERGRG
NLEWKEPPSMRLKARPGNPGFSVLTCSAFSFYPPELQLRFLRNGLAAGTGQGDFGPNSDGSFHASSSLTVKSGDEHHYCCIVQHAGLA
QPLRVELETPAKSSVLVVGIVIGVLLLTAAAVGGALLWRRMRSGLPAPWISLRGDDTGSLLPTPGEAQDADSKDINVIPATA

Fig. S6. Mass spectrometry identification of FcRn expression in cos-7 cells. Mass spectrometry detected peptides of FcRn in cos-7 cells and their sequence coverage. Table of the observed peptides from LC-MS (diaPASEF analysis of the cell lysate digest; gas phase fractionation analyses 1-4 (GPF1-4) of the sample where at least one peptide from the protein was observed) with basic qualitative characteristics provided by DIA-NN application: Prec. Count – number of precursors reported in the given LC-MS run for individual peptides; Lib. Q Value – empirical library Q values, false discovery rate estimates; PEP – posterior probability value, estimate of the local false discoveries in individual analyses.

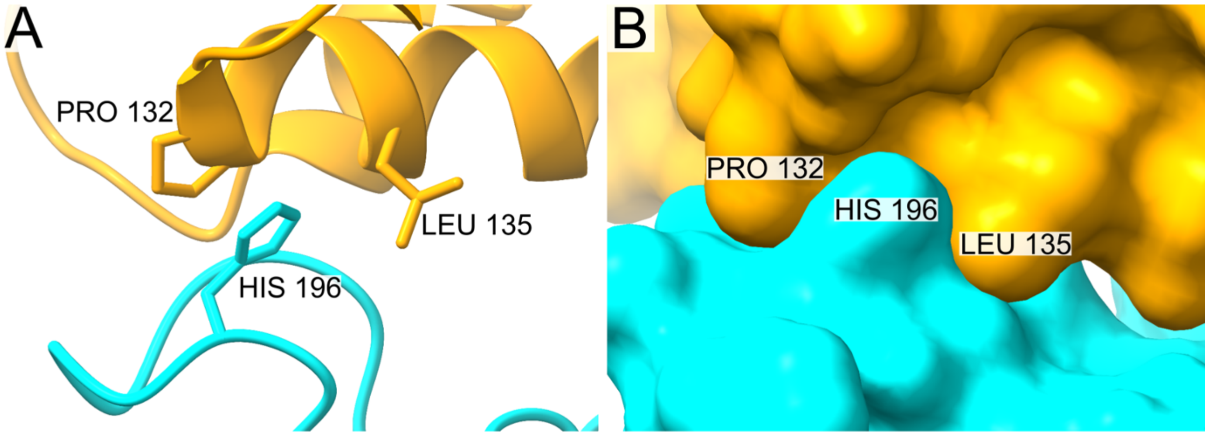

Fig. S7. Interface between FcRn and E18 includes his196 of VP1. (A) Cartoon representation of part of the interface between FcRn (orange) and VP1 (cyan), including the sidechains of pro132 and leu135 of FcRn and his196 of VP1, which are shown in stick representation. **(B)** Molecular surface representation of the same interface as in panel (A).

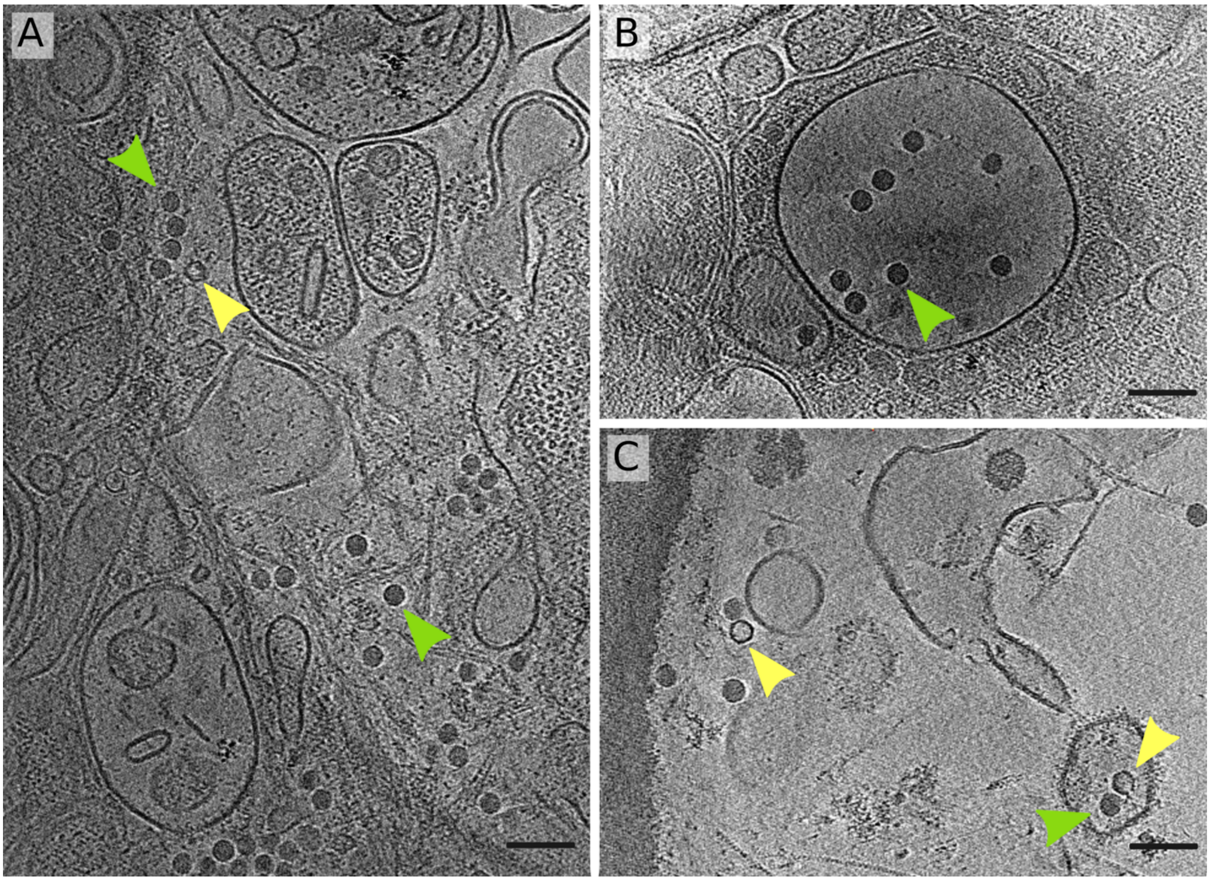

Fig. S8. Bafilomycin A1 treatment of cells reduces number of E18 infecting particles in cos-7 cells that release their genomes. (A-C) Sections of cryo-tomograms of cos-7 cells pre-treated with the bafilomycin A1 for thirty minutes before infection and vitrified 30 minutes after E18 infection. Yellow arrowheads indicate empty capsids, and green arrowheads indicate genome-containing particles of E18. Scale bars represent 100 nm.

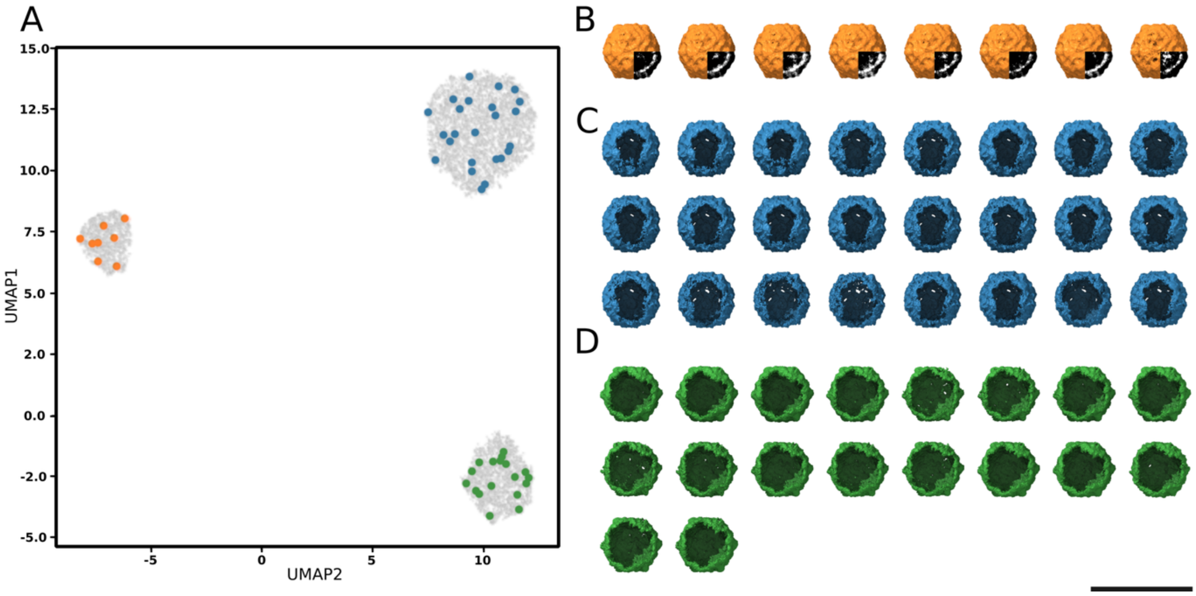

Fig. S9. Structural heterogeneity analysis of E18 particles *in situ*. (A) Latent space representation of the open particles based on their structural features, analyzed using the program CryoDRGN (1, 2). The analysis identified three clusters representing (orange) genome-containing particles with a capsid conformation corresponding to that of the E18 virion but missing the pocket factor, (blue) open particles missing one or two pentamers, and (green) open particles missing three pentamers. **(B-D)** Three-dimensional representations of particles representing selected positions in the latent space, as indicated in panel (A). (B) particles missing one pentamer, (C) particles missing one or two pentamers, (D) particles missing three pentamers. Scale bar indicates 50 nm.

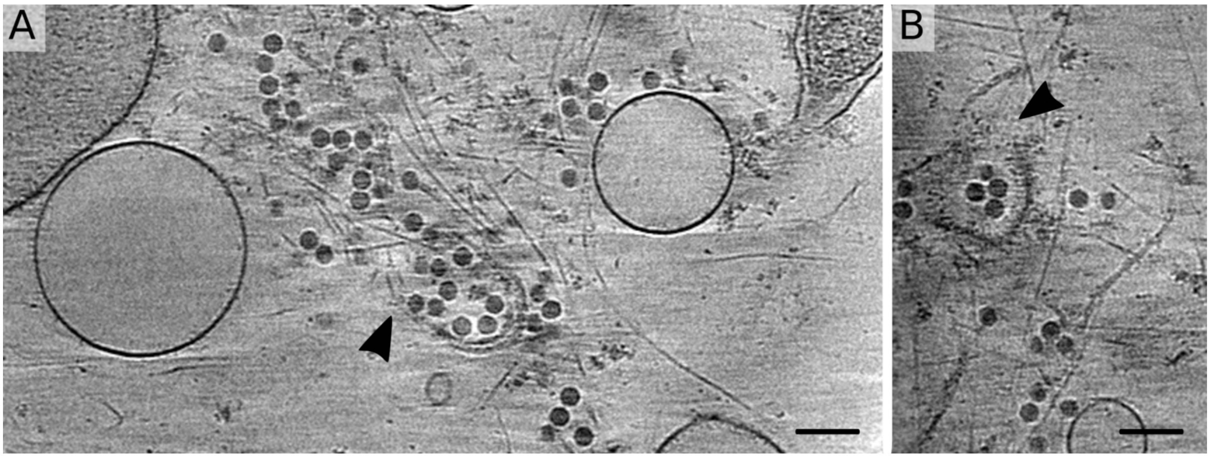

Fig. S10. Release of E18 particles from ruptured endosomes. (AB) Sections of cryo-tomograms of cos-7 cells vitrified 30 minutes post E18 infection. Black arrowheads indicate ruptured endosomes releasing E18 particles into the cytoplasm. Scale bars represent 100 nm.

Supplementary Table S1. Cryo-EM data and structure quality indicators.

Structure	Virion	Virion in situ	Virion in situ	Open particle missing 1 pentamer	Open particle missing 1 pentamer	Open particle missing 2 pentamers	Open particle missing 2 pentamers	Open particle missing 3 pentamers	Open particle missing 3 pentamers	Particle in complex with FcRn	Activated particle	Empty particle
Symmetry	I	C1	I	C1	C5	C1	C2	C1	C3	I	I	I
Nominal magnification	75,000 x	42000 x	42000 x	42000 x	42000 x	42000 x	42000 x	42000 x	42000 x	75,000 x	75,000x	75,000x
Voltage (kV)	300	300	300	300	300	300	300	300	300	300	300	300
Flux on detector (e-/pix sec)	22,05	23,6	23,6	23,6	23,6	23,6	23,6	23,6	23,6	45,6	45,6	45,6
Electron exposure (e-/Å2)	23,4	33,8	33,8	33,8	33,8	33,8	33,8	33,8	33,8	54,5	54,5	54,5
Calibrated pixel size (Å)	1,061	2,08047	2,08047	2,08047	2,08047	2,08047	2,08047	2,08047	2,08047	1,061	1,061	1,061
Number of collected movies	938	1290	1290	1290	1290	1290	1290	1290	1290	4533	4533	4533
Initial particle images	42863	22729	22729	22729	22729	22729	22729	22729	22729	80069	80069	80069
Final particle images	10062	1337	1337	3896	3896	6365	6365	2114	2114	16453	12815	3776
Map resolution at FSC=0.143 (Å)	3,1	9,5	4,3	8,9	6,5	9,6	8,3	18,1	10	2,26	2,44	2,86
**Refinement**												
Map sharpening B factor	-82,8		-220,6		-291,24					-60,97	-66,67	-69,48
Model compositions												
Number of non-hydrogen atoms	6121		6130		57156					7607	6093	5173
Protein residues	770		784		7480					954	659	660
Number of ligands	2									1	0	0
**R.M.S. deviation**												
Bond lengths	0,004		0,002		0,002					0,002	0,002	0,002
Bond angles	0,736		0,44		0,626					0,531	0,445	0,547
**Molprobity validation**												
Molprobity score	2		1,66		1,69					1,5	1,61	1,6
Clashscore	14,31		5,8		8,63					8,51	7,76	5,1
Rotamers												
Favored (%)	99,7		99,56		99,74					99,88	100	100
Poor (%)	0,3		0		0,18					0,12	0	0
**Ramachandran plot**												
Favored (%)	95,14		95,06		96,54					97,84	96,87	95,31
Allowed (%)	4,21		4,94		3,46					2,16	3,13	4,69
Poor (%)	0,65		0		0					0	0	0
**Accession codes**												
PDB	6HBG		9S63		9SPU					9TF0	9TF1	9TF2
EMDB	EMD-0181	EMD-54484	EMD-54622	EMD-54764	EMD-55096	EMD-54510	EMD-54506	EMD-54765	EMD-54763	EMD-55863	EMD-55864	EMD-55865

Supplementary Movie Legend:

Movie S1. Cryo-tomogram and segmentation together with template matching of the cytoplasm of a Cos7 cell, 30 minutes postinfection by E18. The positions and orientations of genome-containing (green) and open E18 particles (yellow), as well as ribosomes (violet) and microtubules (brown), were determined using template matching. Actin filaments (cyan) were segmented manually. The magnified inset in the upper left corner highlights the various particle states in the cell cytoplasm. Scale bars represent 50 nm. The movie corresponds to the tomogram segmentation shown in Fig. 4.

Supplementary references:

1. R. Rangan *et al.*, CryoDRGN-ET: deep reconstructing generative networks for visualizing dynamic biomolecules inside cells. *Nat Methods* **21**, 1537-1545 (2024).
2. E. D. Zhong, T. Bepler, B. Berger, J. H. Davis, CryoDRGN: reconstruction of heterogeneous cryo-EM structures using neural networks. *Nat Methods* **18**, 176-185 (2021).
